# Supplementary material for: Three-Dimensional Printed Carbon Black/PDMS Composite Flexible Strain Sensor for Human Motion Monitoring
Source: Micromachines (Basel). 2022 Aug 2;13(8):1247. doi: 10.3390/mi13081247 (PMC9416005; doi:10.3390/mi13081247)
Supplement: Supplementary file 1 [file micromachines-13-01247-s001.zip › micromachines-1855852-supplementary.pdf]

Support material

3D printed carbon black/PDMS composite flexible strain sensor for human motion monitoring

Haishan Lian [1], Ming Xue [2], Kanglin Ma [1], Deyun Mo [1], Zaifu Cui [1], Lei Wang [1,*] and Xiaojun Chen [1,*]

1 School of Mechanical and Electronic Engineering, Lingnan normal university, Zhanjiang, China

2 Dongguan Technician College, Dongguan, China

* Correspondence: wanglei1999-1999@163.com (L.W); chxj@lingnan.edu.cn (XJ.C)

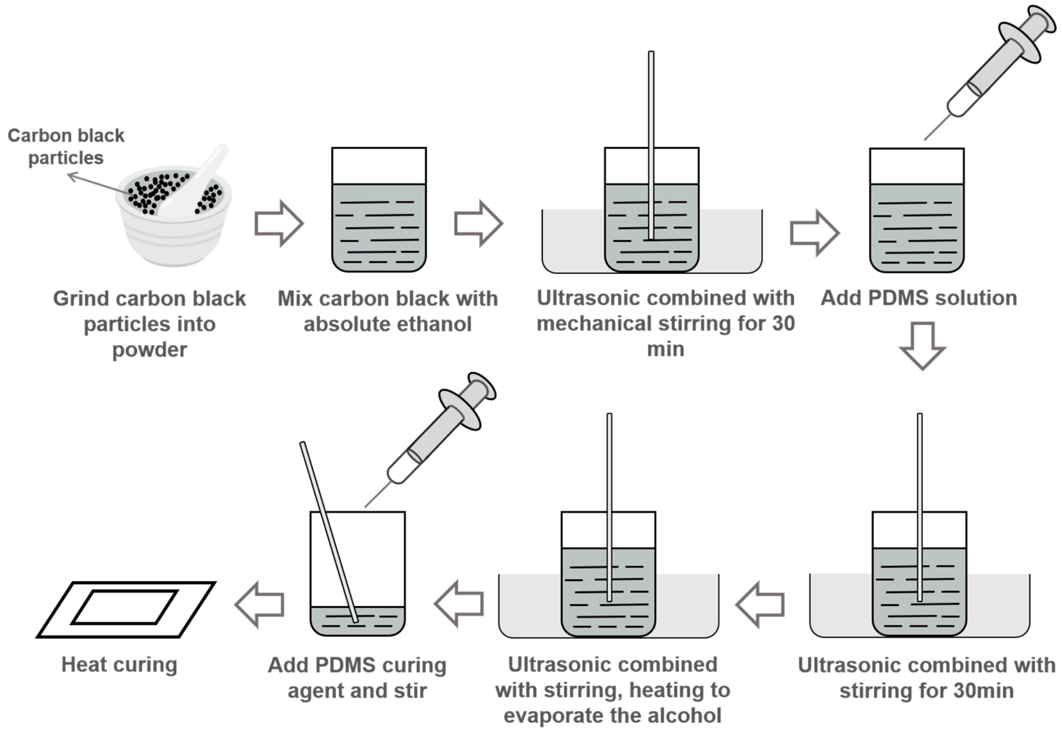

Figure S1: Preparation process of carbon black/PDMS composites
